# Sural Nerve Graft Donor-Site Morbidity in Nipple–Areola Complex Sensory Reinnervation: A Prospective Cohort Study

**DOI:** 10.3390/medicina62101864

**Published:** 2026-09-26

**Authors:** Jānis Lapiņš, Beatriz Soares Domingues Polita, Linda Kalniņa, Michal Grucki, Dzintars Ozols, Ansis Ģīlis, Arvīds Irmejs, Kristaps Blūms, Gundega Ķauķe, Jānis Gardovskis, Jeļena Maksimenko

**Affiliations:** 1 Department of Doctoral Studies, Rīga Stradiņš University, LV-1007 Riga, Latvia; 2 Pauls Stradiņš Clinical University Hospital, Pilsoņu iela 13, LV-1002 Riga, Latvia; 3 Universitätsklinikum Gießen (UKGM), Klinikstraße 33, 35392 Gießen, Germany; 4 Faculty of Medicine, Rīga Stradiņš University, LV-1007 Riga, Latvia; 5 Department of Pediatric Surgery, Children’s Clinical University Hospital, LV-1004 Riga, Latvia; 6 Institute of Oncology and Molecular Genetics, Rīga Stradiņš University, LV-1007 Riga, Latvia; 7 Department of Surgery, Rīga Stradiņš University, LV-1007 Riga, Latvia

**Keywords:** sural nerve, nerve graft, donor-site morbidity, nipple–areola complex, sensory reinnervation, electroneurography, two-point discrimination, monofilament test, breast reconstruction

## Abstract

*Background and Objectives*: Sural nerve grafts are increasingly used to restore sensation after reconstructive surgery, including nipple–areola complex (NAC) reinnervation following nipple-sparing mastectomy, but donor-site morbidity has not been characterized using combined objective and patient-reported measures. We quantified this morbidity prospectively. *Materials and Methods*: Ten women who had undergone mastectomy with direct-to-implant reconstruction and NAC reinnervation using a sural nerve graft were assessed by photographic mapping of the donor-site deficit, the FootQ symptom and scar-satisfaction questionnaire (preoperatively, 6 months, 1 year), sural nerve conduction studies (NCS) at 12 months (at 10 months in one patient), and bedside Semmes–Weinstein monofilament and two-point discrimination (2PD) testing. Spearman correlation, Wilcoxon signed-rank, and Friedman tests were used. *Results*: Harvest length and deficit area were not significantly associated (ρ = −0.588, *p* = 0.074; bootstrap 95% CI −0.90 to +0.08); with ten patients, the analysis was underpowered and neither establishes nor excludes an association. Deficit area was strikingly variable (coefficient of variation 56%) and did not differ by neurotoxic chemotherapy exposure (five per subgroup). At 1 year, seven of nine patients (78%) had an unrecordable sural sensory action potential, yet monofilament thresholds were normal in every evaluable patient, and 2PD did not differ between limbs. FootQ symptoms increased from the preoperative assessment to 6 months (composite 1.34 to 2.19; *p* = 0.047), then remained mild and unchanged to 1 year (all *p* ≥ 0.50); scar satisfaction remained high. *Conclusions*: Sural graft harvest produces a highly variable and often electrophysiologically persistent donor-site sensory deficit that bedside testing and patient-reported outcomes did not detect at 1 year; because those tests used fixed anatomical sites, the discordance may reflect the territory assessed as much as test sensitivity or peripheral compensation. This small cohort neither establishes nor excludes an association between harvest length and the residual deficit, and the donor site remained clinically well tolerated.

## 1. Introduction

Mastectomy with immediate implant reconstruction often preserves the breast contour but not its sensation. Complete or partial anesthesia of the nipple–areola complex (NAC) is a common, patient-relevant, and comparatively understudied shortcoming of reconstruction. One way to address it is NAC sensory reinnervation with an autologous nerve graft coapted proximally to an intercostal nerve and distally to the areolar nerve stump. We previously described this technique using the sural nerve as graft material in a case report [1], in which we noted that donor-site (lower-leg) morbidity had not been assessed systematically in this setting and stated our intention to expand the cohort for an adequately powered follow-up study.

The sural nerve is the most commonly used autologous nerve graft donor, valued for its accessible and constant anatomy, its expendable, purely sensory function, and a length of up to 30–40 cm [2,3]; its use as graft material dates back decades [4]. Its donor-site morbidity has been examined in several studies of varying methods and follow-up. In a systematic review of nine studies (240 patients), Bamba et al. [5] reported sensory loss at the donor site in 87.2% of patients, with the affected area generally decreasing over time. A larger pooled analysis by Ducic et al. (12 studies, 478 procedures) found a comparable 92.9% rate of sensory deficit, with chronic pain in 19.7% and an impact on daily life in only 5.0% [6]; the most recent systematic review reached the same overall conclusion [7]. Tada et al. [8] followed 13 patients for a mean of 29.5 years and found that sensory disturbance and movement difficulty tended to improve, with little effect on daily activities and high satisfaction. Hallgren et al. [9] surveyed 41 patients reconstructed 1.2 to 39 years earlier and likewise reported favorable subjective outcomes despite an objective deficit. Studies with the longest horizons agree: IJpma et al. re-examined patients up to 34 years after harvest and found that both the size and severity of the sensory-loss area improved markedly over time [10], echoing earlier telephone and questionnaire surveys that had already described substantial subjective recovery [11,12].

Only two studies report a photographically measured deficit area in square centimeters. Ng et al. followed 20 patients and found the anesthesia area fell from 65 cm^2^ at 1 month to 15 cm^2^ at 1.5 years, stabilizing at a mean of 12 cm^2^ (anesthesia) and 55 cm^2^ (combined deficit) by 2 years [13]. Martins et al. similarly reported a shrinking deficit over the first postoperative year that correlated with harvest length [14]. Both measured the deficit serially during its early, most rapidly resolving phase; our cross-sectional measurement at 12 months postoperatively instead captures the deficit after most of that early resolution, which limits direct numerical comparison and is the more likely explanation for any apparent difference in size or in the relationship with harvest length. Objective, instrumented assessment otherwise remains uncommon; even endoscopic-harvest series that add formal sensory testing are the exception [15]. Combined together, the literature suggests that sural harvest produces a common but well-tolerated donor-site deficit, but it rests largely on questionnaires and simple bedside tests rather than on a combined photographic, electrophysiological, and patient-reported assessment.

Our aim was to describe sural donor-site morbidity in a 10-patient cohort after breast NAC reinnervation, using four complementary methods: photographic deficit-area mapping, the FootQ symptom and satisfaction questionnaire, sural nerve conduction studies (NCS) at 12 months postoperatively, and bedside sensory testing (Semmes–Weinstein monofilament threshold and two-point discrimination). Specifically, we asked whether harvest length predicts the deficit area, and whether objective electrophysiology and simple bedside or self-reported measures give a consistent or a divergent picture of donor-site recovery.

## 2. Materials and Methods

### 2.1. Patients and Surgical Technique

Ten consecutive patients who underwent bilateral mastectomy with direct-to-implant breast reconstruction and NAC sensory reinnervation with an autologous sural nerve graft were enrolled prospectively at the time of surgery, between 19 December 2023 and 6 October 2025. Assessments were scheduled before surgery, at 6 months and at 12 months postoperatively. Nine patients completed the final assessment 12 months after surgery; in the patient operated on last (Patient 8), the final assessment, including the nerve conduction study, was performed 10 months after surgery, because follow-up had to be fitted to the patient’s availability within the data-collection period of the study. The final assessment therefore took place at a median of 12 months (range 10–12) after surgery, and no patient was assessed later than 12 months. All surgeries were performed after Institutional Review Board approval (18 January 2023); no patient was enrolled or treated before this date, and no eligible patient was excluded. Mean age at surgery was 41.7 years (SD 8.6; median 41.5; range 33–58). Patient 1 in Table 1 is the case previously reported in [1]; her donor-site outcomes are included here as part of the expanded cohort that was anticipated. In every case, the graft was coapted proximally to an intercostal nerve and distally to the areolar undersurface. The sural nerve was harvested using the classic stepladder technique—a series of short, staggered transverse incisions along its subcutaneous course on the posterior calf, with the intervening skin bridges connected under direct vision. NAC reinnervation was bilateral in eight patients and unilateral in two of the earliest patients in the series (Patients 1 and 2), in whom only one nipple–areola complex was reinnervated. In the eight patients reinnervated bilaterally, the minimum required graft length was determined empirically and individually before surgery by measuring the surface distance from the nipple to the anterior axillary line on each breast, and the nerve was harvested from a single donor leg to a length sufficient to supply both sides. In the two patients reinnervated on one side only, no bilateral requirement was measured; the graft was harvested at a length judged intraoperatively to be sufficient for a tension-free coaptation on the treated side, and these two patients account for the two shortest harvests in the cohort (12 and 14 cm). In every patient, whether reinnervated on one side or on both, the length harvested exceeded the length ultimately coapted, because a safety margin is retained as a matter of routine: additional graft cannot be obtained once the donor site has been closed. Harvested length was measured with a ruler laid flat against the excised, unstretched nerve segment, from its proximal to its distal end. All procedures were performed by a single surgeon at Pauls Stradiņš Clinical University Hospital. Harvested nerve length ranged from 12 to 26 cm (mean 21.4, SD 4.8). Five patients had received neoadjuvant or adjuvant neurotoxic chemotherapy, in each case a taxane-based regimen with or without platinum. One of them had generalized peripheral neuropathy confirmed on nerve conduction studies; chemotherapy-induced neuropathy is plausible given her exposure [16], but cannot be confirmed because no electrophysiological study was performed before chemotherapy (Section 4.5).

### 2.2. Photographic Sensory-Deficit Area Mapping

Sensory-deficit zones on the lower leg were outlined on the skin and photographed against a metric ruler for scale. Zone boundaries were determined by monofilament-guided mapping: Semmes–Weinstein monofilament testing (Jamar Monofilaments; Performance Health Supply, Inc., Cedarburg, WI, USA) was performed in a radial pattern outward from the harvest incision, and the skin was marked at the point where the threshold response changed—from normal to reduced sensation for the outer (blue) boundary, and from any perceptible response to none for the inner (red) boundary—before photography. Bedside mapping was performed by co-author L.K. for all 10 patients, independent of the corresponding author, to limit between-patient examiner variability; formal inter- or intra-observer reliability of the boundary-drawing step was not tested, which we note as a limitation (Section 4.5). Two zones were distinguished: a blue zone for any altered but preserved sensation (hypoesthesia or dysesthesia) and a red zone for complete anesthesia. A red zone was present in 3 of the 10 patients. Photographs were taken with a Nikon D810 full-frame camera (Nikon Corporation, Tokyo, Japan) and a fixed 105 mm lens (confirmed from the EXIF metadata). Areas were obtained by digital image segmentation, converting pixels to square centimeters with a photograph-specific calibration coefficient (240–614 px/cm across the series). Automated contour-and-fill algorithms were used, with manual closure of the boundary in the few photographs where it was not fully continuous. Where possible, calibration was cross-checked against at least two independent ruler references. Segmentation and area calculation were carried out with the assistance of an AI tool (Claude Opus 4.8; Anthropic, PBC, San Francisco, CA, USA), which generated and executed the image-processing code in Python 3.11 (Python Software Foundation, Wilmington, DE, USA) using NumPy 2.4, SciPy 1.17 and Pillow 12.2, under the direct supervision of the author, with manual verification of every result. The deficit boundary itself was always determined by the examiner on the patient’s skin, never by the AI tool; the AI tool’s role was limited to converting the photographed, examiner-drawn boundary into a filled pixel mask and computing area from the examiner-supplied calibration (blue zones, automated color segmentation with manual closure where needed; red zones, manual polygon tracing, since automated color detection did not reliably separate red-marked skin from unmarked skin). Every segmentation was visually verified against the source photograph by the corresponding author, who was not involved in the bedside mapping, before the area was accepted.

The uncertainty of each area was estimated in two ways: by comparing the calibration coefficient with a second, independently derived coefficient (a different ruler reference or a parallel scale) and recomputing the area, and by checking how far the segmented boundary moved when the contour threshold was changed slightly (its plateau stability). The larger uncertainties (±15–20%) apply to curved surfaces such as the ankle and heel, where a two-dimensional photograph underestimates the true surface area, and to images in which ruler parallax made the two calibration coefficients diverge. The smallest uncertainty (±12%) applies to flat surfaces measured against a single, well-confirmed scale.

### 2.3. FootQ Questionnaire

Donor-site symptoms and scar satisfaction were recorded with FootQ, a non-validated symptom and satisfaction questionnaire designed by our group for this study (not a previously published, validated instrument), administered before surgery, at 6 months, and at 1 year. The symptom score is the mean of eight items—pain, numbness, numbness-related discomfort, mechanical allodynia, thermal allodynia, hyperesthesia, paresthesia, and stiffness—each rated from 1 to 5, with higher scores indicating worse. Scar satisfaction (item Q9) is scored in the opposite direction, from 1 (very dissatisfied) to 5 (completely satisfied); it was analyzed on its own and never added to the symptom score. Because no donor-site scar exists before surgery, the pre-operative response to this item cannot be interpreted as scar satisfaction in the sense intended; it was therefore not analyzed, is not reported numerically, and is not displayed in Figure 1. All scar-satisfaction results and comparisons are restricted to the 6-month and 1-year timepoints.

### 2.4. Electroneurography (NCS)

Sural sensory nerve conduction studies were performed in 9 of the 10 patients at the final assessment: 12 months postoperatively in eight of them and 10 months postoperatively in one (Section 2.1). Sensory nerve action potentials (SNAPs) were recorded on both legs, the operated donor limb being compared with the contralateral limb as an internal control. One report was unavailable.

### 2.5. Bedside Sensory Tests

The Semmes–Weinstein monofilament threshold (log-force, with 1.65 the most sensitive normal value) was measured at a foot site and an ankle site at several time points. Two-point discrimination (2PD) was tested at four sites on each leg, on the operated and the control side. The foot and ankle monofilament sites and the four 2PD sites per leg were fixed anatomical landmarks used consistently across all patients and timepoints for longitudinal comparability; they were not individually selected to fall inside each patient’s own photographically mapped deficit zone, which varies in location and extent between patients. Responses were coded according to the study protocol as 0 (no point perceived, anesthesia), 1 (one point perceived, impaired discrimination), and 2 (two points perceived, normal).

### 2.6. Statistics

Descriptive statistics (mean, SD, median, range, coefficient of variation) and Spearman rank correlation with a bootstrap 95% confidence interval were used to relate harvest length to deficit area. Chemotherapy subgroups were compared with the Mann–Whitney U and Fisher’s exact tests. Longitudinal FootQ data were analyzed with the Wilcoxon signed-rank and Friedman tests. For the symptom composite score, which is the mean of eight ordinal items, a paired *t*-test was computed alongside the Wilcoxon signed-rank test as a sensitivity check (Supplementary Table S1); the non-parametric result was treated as primary throughout. All tests were two-tailed with α = 0.05. All statistical analyses were performed in Python 3.11 with NumPy 2.4 and SciPy 1.17. Given the sample size, the results should be read as exploratory rather than confirmatory.

## 3. Results

### 3.1. Cohort

The cohort is summarized in Table 1. Harvest length ranged from 12 to 26 cm. A blue (altered-sensation) zone was present in every patient, and a red (complete-anesthesia) zone in three. NCS data were available for nine patients.

### 3.2. Photographic Sensory-Deficit Area Analysis

Descriptive statistics for harvest length and the mapped areas are given in Table 2. The altered-sensation (blue) area varied far more than harvest length: its coefficient of variation was 56%, about two and a half times that of harvest length (22%), and it spanned 6.1 to 37.2 cm^2^—a 6.1-fold range against the 2.2-fold range of harvest length. The complete-anesthesia (red) area, present in only three patients, was the most variable of all.

Harvest length and altered-sensation area were negatively but not significantly correlated (ρ = −0.588, *p* = 0.074), and the bootstrap 95% CI [−0.90, +0.08] crossed zero, so neither the strength nor even the direction of the association can be stated with confidence. With ten patients, this analysis had low power, and the interval is compatible both with no association and with a moderate-to-strong association in either direction. Excluding the patient with generalized polyneuropathy weakened the correlation further (ρ = −0.420, *p* = 0.260). Correlations with the total deficit area and with the red area are given in Table 3, and a leave-one-out sensitivity analysis of the primary correlation in Table 4.

**Table 3:** Spearman correlations between harvest length and the mapped sensory-deficit areas.

Correlation	n	Spearman ρ	95% CI	*p*
Harvest length vs. altered-sensation (blue) area	10	−0.588	[−0.90, +0.08]	0.074
Harvest length vs. altered-sensation area, excluding the polyneuropathy patient	9	−0.420	—	0.260
Harvest length vs. total deficit area	10	−0.330	—	0.352
Harvest length vs. complete-anesthesia (red) area	10	+0.310	—	0.383

**Table 4:** Sensitivity analysis of the correlation between harvest length and altered-sensation (blue) area (row 1 of Table 3). Every leave-one-out estimate is based on n = 9; the row in which two patients are omitted is an additional leave-two-out check (n = 8).

Patient(s) Removed	n	Spearman ρ	*p*
Patient 3 (polyneuropathy, longest harvest, smallest area)	9	−0.420	0.260
Patient 4 (shortest of the long harvests, largest area)	9	−0.511	0.160
Patient 8 (assessed at 10 months; second largest area)	9	−0.757	0.018
Patients 3 and 4	8	−0.273	0.513
Full leave-one-out series (each patient removed in turn)	9	−0.42 to −0.76	0.02–0.26

Across the full leave-one-out series (n = 9 for every estimate), the coefficient ranged from −0.42 to −0.76 and the *p*-value from 0.02 to 0.26. The point estimate is therefore materially dependent on which one or two patients are included; the correlation is not stable to the removal of a single patient and should be read as hypothesis-generating only. It supports neither the conclusion that harvest length influences the deficit area nor the conclusion that it does not. The coefficient is most sensitive to two patients: removing Patient 3 weakens it to ρ = −0.420 (*p* = 0.260), whereas removing Patient 8 strengthens it to ρ = −0.757 (*p* = 0.018). Patient 8 is also the one patient whose assessment took place at 10 rather than 12 months (Section 2.1 and Section 4.5).

#### Discordant Cases and Chemotherapy Exposure

Two patients show how loosely harvest length and deficit area are linked. The patient with the largest altered-sensation area (37.2 cm^2^) had a relatively short, 19 cm harvest and no chemotherapy, whereas the patient with the longest harvest (26 cm) had the smallest area (6.1 cm^2^)—and was also the one with documented generalized peripheral neuropathy. This pair largely accounts for the negative direction of the correlation. In a cohort of this size, two discordant patients dominate the rank statistic, so these observations describe the variability encountered rather than supporting any inference about the influence of harvest length in either direction. In exploratory subgroup comparisons, neither the altered-sensation area (Mann–Whitney U, *p* = 0.84) nor the total deficit area (*p* = 0.10) differed between patients with and without chemotherapy, and the red zone (3 of 10 patients) was not associated with chemotherapy exposure (Fisher’s exact, *p* = 1.0). With only five patients in each chemotherapy subgroup, these comparisons are underpowered; as with the harvest-length correlation, the absence of a significant difference does not establish that chemotherapy has no effect on the deficit area.

### 3.3. FootQ: Symptom and Satisfaction Trajectory

FootQ outcomes are shown in Figure 1. The mean symptom composite score rose from 1.32 before surgery to 2.36 at 6 months and was essentially unchanged at 1 year (2.42), indicating mild donor-site symptoms that did not worsen during later follow-up. Scar satisfaction was high and stable across the two postoperative timepoints at which a scar existed to be rated (3.62 at 6 months, 3.80 at 1 year; Supplementary Table S2). No preoperative scar-satisfaction value is reported or displayed, because no donor-site scar existed at that assessment (Section 2.3).

The overall symptom trajectory differed significantly across the three assessment points (Friedman χ^2^ = 7.10, *p* = 0.029, n = 8), driven by a significant early postoperative rise: the composite score increased from 1.34 before surgery to 2.19 at 6 months (Wilcoxon *p* = 0.047). No further change occurred between 6 months and 1 year (2.19 to 2.32; all item-level *p* ≥ 0.50, Supplementary Table S2). The clinically relevant pattern is therefore an early rise in donor-site symptoms during the first postoperative months, which then plateaus at a mild, stable level rather than continuing to worsen or to resolve. None of the eight symptom items, nor scar satisfaction, changed significantly between 6 months and 1 year (all *p* ≥ 0.50, Wilcoxon; Supplementary Table S2). Sensitivity and exploratory analyses of the symptom composite score are provided in Supplementary Table S1. The 6-month-to-1-year comparison remained non-significant whether all paired observations were included (2.36 to 2.57; paired *t*-test *p* = 0.617, Wilcoxon *p* = 0.781) or one atypical 6-month response was excluded (2.53 to 2.37; *p* = 0.359 and *p* = 0.281). The exploratory comparisons in Supplementary Table S1 are consistent with this early rise (preoperative vs. 6 months, *p* = 0.047 Wilcoxon and 0.058 paired t-test; preoperative vs. 1 year, *p* = 0.078 and 0.073), which was not followed by any further worsening. Means differ slightly between the descriptive figures above and the paired analyses because each paired comparison includes only patients with data at both, or all three, of the relevant assessments. Denominators vary across FootQ comparisons because of ordinary incomplete questionnaire return: Patient 1 did not return a FootQ questionnaire at the 6-month visit (reducing 6-month-versus-1-year comparisons to n = 9), and a small number of individual items were left blank by individual patients at a given visit (e.g., Patient 4 did not answer the scar-satisfaction item at 6 months, n = 8 for that comparison).

### 3.4. Electroneurography (NCS) at 12 Months Postoperatively

NCS data were available for nine patients. On the operated side, the sural SNAP was unrecordable in 7 of 9 (78%); the remaining two retained recordable but markedly reduced amplitudes of 0.86 and 2.9 µV. The contralateral response was recordable in every patient, confirming the operated limb as the site of conduction loss. One patient had generalized sensorimotor polyneuropathy, and one had incidental findings suggesting an L5 radiculopathy. A year after harvest, NCS still showed objective sural sensory impairment on the donor side in most patients (Table 5).

### 3.5. Foot Sensitivity: Semmes–Weinstein Monofilament

Monofilament thresholds at the foot and ankle were variable and raised in the early months after surgery—foot-site means of 1.85 to 2.30 and ankle-site means of 1.65 to 1.98 through the first 6 months, with individual values as high as 4.17. By 1 year, they had normalized: every evaluable patient reached 1.65 at both sites, with no spread (Table 6).

This contrasts sharply with the NCS findings at the same point. Of the seven patients with both a 1-year monofilament result and an available NCS, six had an unrecordable sural SNAP, yet all seven had a normal monofilament threshold. In this cohort, monofilament testing did not detect the persistent electrophysiological deficit that NCS demonstrated; possible reasons are taken up in the Discussion.

### 3.6. Two-Point Discrimination (2PD)

Two-point discrimination was flat across follow-up and gave no operated-side signal. The proportion of sites with normal discrimination on the operated leg tracked the control leg at every time point, including 1 year (53% on each side), and no anesthesia was recorded at 1 year (Table 7). As with the monofilament test, 2PD did not detect a difference attributable to the donor-side deficit demonstrated by NCS.

## 4. Discussion

This study brings together four complementary methods for assessing sural donor-site morbidity in a single cohort. Its central observation is a clear discordance between them: photographic mapping and NCS both show an objective, persistent deficit, while bedside testing at fixed anatomical sites did not detect it at 1 year, though we cannot fully separate genuine recovery, test insensitivity, and spatial mismatch between the fixed test site and each patient’s mapped deficit zone as explanations.

### 4.1. Principal Findings

Three findings stand out. First, we detected no significant association between harvest length and the size of the visible deficit; the point estimate was negative, with longer grafts tending to smaller mapped areas, but it was unstable in leave-one-out analysis, so at this sample size neither an association nor its absence can be established. Harvest length in this cohort was not chosen experimentally: in the eight patients reinnervated bilaterally it followed each patient’s own chest-wall dimensions, and in the two patients reinnervated on one side only it followed an intraoperative judgment of sufficiency. In both situations, the harvested length also included a routine safety margin over the length actually coapted. Neither rule is tied to the anatomy of the donor nerve territory, which may partly explain why harvest length did not track the donor-site deficit area. Second, a year after surgery, most patients still had no recordable sural sensory response, so the deficit is real and, in conduction terms, often permanent. Third, that deficit was not detected by the bedside tests at the sites examined, and was little reflected in what the patients reported—monofilament thresholds were normal in everyone tested at 1 year, 2PD could not separate the operated from the control leg, and FootQ symptoms rose significantly from before surgery to 6 months and then remained stable, at a mild level, through 1 year, while scar satisfaction remained high.

### 4.2. Comparison with Previous Literature

These results fit the existing, largely subjective literature while adding a quantitative dimension to it. Systematic reviews put donor-site sensory loss at 87–93% of patients, with chronic pain in roughly one in five [5,6,7], and the long-term series agree that both the area and the severity of the deficit recede over the years that follow [8,10]. Our cohort shows the same well-tolerated morbidity. The sharpest contrast with prior work concerns harvest length. Martins et al., one of two earlier prospective studies to map the deficit serially, found that the sensory-loss area correlated with the length of nerve harvested [14]; we did not reproduce that association, and observed a non-significant tendency in the opposite direction. The most likely explanation is method and timing: Martins et al. measured area by clinical examination during the first postoperative year, when the deficit is largest and still resolving, whereas our cross-sectional mapping at 12 months reflects the settled result. Whether individual anatomy and reinnervation capacity outweigh graft length at that stage cannot be determined from ten patients; our data show only that an effect of graft length was not detectable in this cohort. Martins et al. and the other serial study, Ng et al. [13], are in fact the exceptions that did report patient-level deficit areas in square centimeters; both measured during the early, most rapidly resolving phase, so their values are not directly comparable to our cross-sectional measurements at 12 months, though the wide variability we measured—a more than sixfold range—matches the heterogeneity they describe.

### 4.3. Discordance Between Objective and Clinical Assessments

The most clinically useful finding is the gap between NCS and the bedside tests at 1 year. Six of seven patients with both assessments had lost sural conduction entirely, yet all seven had a normal monofilament threshold. This is a discordance between two measurements; by itself it does not identify a cause, and several non-exclusive explanations are compatible with it. The sural territory at the foot and ankle overlaps with the superficial peroneal, tibial, and saphenous nerves, so if a test point falls outside an exclusively sural area, neighboring nerves can carry the sensation and mask the loss. The two tests also measure different things: the monofilament assesses touch-pressure threshold over a broad receptive field served by large Aβ fibers, whereas NCS measures conduction in the sural trunk itself. Because harvest is a complete transection rather than a partial injury, collateral reinnervation from adjacent nerves could in principle restore the touch threshold even though the sural trunk never conducts again; anatomical observations are consistent with this possibility, in that removing the entire sural nerve still leaves nociception preserved over the most affected areas and produces no long-term pain [17]. Our data, however, do not test this mechanism and provide no direct evidence for it. Finally, the mapped deficit is spatially limited (mean 18.6 cm^2^, with fairly discrete borders), so a standard foot or ankle test point may simply lie at its edge or beyond it. The same reasoning applies to 2PD, which, in addition, reflects innervation density rather than threshold and carries a high baseline variability (only 56–64% normal responses before surgery), leaving it poorly placed to detect a small deficit at sites with overlapping innervation. However, whether 2PD is useful as a donor-site screening test cannot be judged from these data: both its high baseline variability and the use of fixed, not individually sural-specific, test sites would obscure a true deficit, and answering that question would require a study in which 2PD is tested at individually mapped sites within the deficit zone. Because all bedside testing was performed at fixed anatomical landmarks rather than at points verified to lie inside each patient’s mapped zone, a difference in the anatomical territory assessed is at least as plausible an explanation for the discordance as either peripheral compensation or limited test sensitivity. We therefore report the discordance as an observation and do not attribute it to any one of these mechanisms.

### 4.4. Clinical Implications

Two implications follow. The choice of assessment method strongly shapes what is concluded: a study relying on questionnaires or on bedside tests at fixed anatomical sites would have recorded near-complete recovery and would not have detected the persistent deficit shown by mapping and NCS, so the method should be matched to the question. How much of this reflects genuine insensitivity of the bedside tests and how much the choice of test site cannot be resolved here (Section 4.5). For the patient in front of the surgeon, the message is reassuring but should be honest—a measurable, often electrophysiologically permanent sensory deficit at the donor site is expected, yet in this cohort, and in the wider literature, it rarely causes meaningful functional trouble or dissatisfaction. Harvest length did not show a consistent relationship with deficit size in this exploratory analysis, and the wide confidence interval means this should not yet be treated as an established finding. It offers no more than provisional reassurance that graft length can continue to be chosen primarily to suit the recipient defect; it is not evidence that longer harvests are inconsequential for the donor site, and larger studies are needed before that conclusion can be drawn. Where donor-site sensation is a particular concern, emerging methods such as end-to-side neurorrhaphy of the distal sural stump to the superficial fibular nerve aim to restore dorsolateral foot sensation without sacrificing the graft, although for now these remain anatomical and experimental [18].

### 4.5. Limitations

The main limitation is the cohort size (n = 10), which limits the power of the correlation and subgroup analyses; the *p*-values and confidence intervals should be read accordingly. Harvest length, the exposure variable in that correlation, was also not determined by a single rule across the cohort: the two shortest harvests came from the two patients reinnervated on one side only, in whom the length was judged intraoperatively rather than derived from a bilateral requirement. This heterogeneity adds variability to an exposure whose correlation with deficit area is already unstable in leave-one-out analysis, and is a further reason to read that correlation as hypothesis-generating. This is offset to some degree by the depth of assessment, with each patient characterized anatomically, electrophysiologically, clinically, and by self-report. Photographic mapping has its own constraints: the areas are two-dimensional estimates of sensation on curved surfaces, and calibration and manual boundary definition add some measurement uncertainty—factors that affect absolute areas more than the central finding of wide between-patient variability. The study was single-center, though it followed a standardized technique, and multicenter data would help confirm that these patterns generalize. NCS was not performed before surgery for any patient; the contralateral limb served as an internal control, but a between-limb comparison is not equivalent to a true preoperative baseline and cannot fully exclude pre-existing, subclinical asymmetry in the donor limb. This limits interpretation of postoperative nerve-conduction findings more generally, and most directly limits interpretation of etiology in the patient with generalized polyneuropathy, in whom a chemotherapy-related cause remained possible but unconfirmed [16]. NCS was also missing for one patient at the 12-month assessment. One patient (Patient 8), who was operated on last, was assessed at 10 months rather than at 12; because both the mapped deficit and the extent of reinnervation continue to change during the first postoperative year, her deficit area may be somewhat larger, and her nerve conduction somewhat less recovered, than a true 12-month measurement would have been. Both effects are conservative with respect to our findings rather than favorable to them: hers is the second largest area in the cohort and, combined with a harvest length of 24 cm (the cohort median, exceeded by only one patient), it is the observation that most attenuates the negative harvest-length correlation (excluding her, ρ = −0.757, *p* = 0.018), and her sural sensory response was one of only two that remained recordable. No patient was assessed later than 12 months. Because the bedside test sites were fixed anatomical landmarks rather than points individually chosen within each patient’s mapped deficit zone, the discordance between NCS and bedside testing cannot be interpreted as a strict same-location comparison; part of it may reflect spatial mismatch between the fixed test point and the mapped zone rather than true insensitivity of monofilament or 2PD testing at the affected site itself. This limits how far the bedside-versus-mapping discordance can be generalized. The chemotherapy-exposed subgroup was small. FootQ is an author-designed instrument without formal psychometric validation or an established minimal clinically important difference; no validated questionnaire specific to lower-leg or calf nerve-graft donor-site morbidity existed at the time of study design. FootQ results should therefore be interpreted as descriptive and exploratory rather than as validated patient-reported outcome data. Finally, follow-up of this cohort is continuing, and further assessments will show whether the mapped areas, bedside findings, conduction results, and reported symptoms hold steady or shift beyond 1 year.

## 5. Conclusions

In this cohort, harvest length showed no clear relationship with the mapped donor-site sensory-deficit area (ρ = −0.588, 95% bootstrap CI −0.90 to +0.08); leave-one-out analysis shows this estimate is driven substantially by two discordant patients, so these data neither establish nor exclude an association between harvest length and the residual sensory deficit. What the data do show clearly is that deficit area varies far more between patients than harvest length does, which is compatible with a multifactorial rather than a length-dependent process but does not establish one. A year after surgery, nerve conduction studies still showed objective sural sensory loss in most patients, yet monofilament thresholds were normal in every evaluable patient—including those with concurrent conduction loss—and two-point discrimination could not distinguish the operated from the control limb. Simple bedside testing at fixed anatomical sites therefore did not detect the persistent electrophysiological deficit that follows sural harvest; whether this reflects the different anatomical territory assessed, limited sensitivity of those tests, or compensation by adjacent nerves cannot be determined from these data.

Patient-reported symptoms rose significantly in the early postoperative period and then remained mild and stable through 1 year, and scar satisfaction was high throughout. Taken together, sural nerve donor-site morbidity after NAC sensory reinnervation is objectively measurable and often electrophysiologically lasting, but clinically well tolerated.

## Figures and Tables

**Figure 1:**
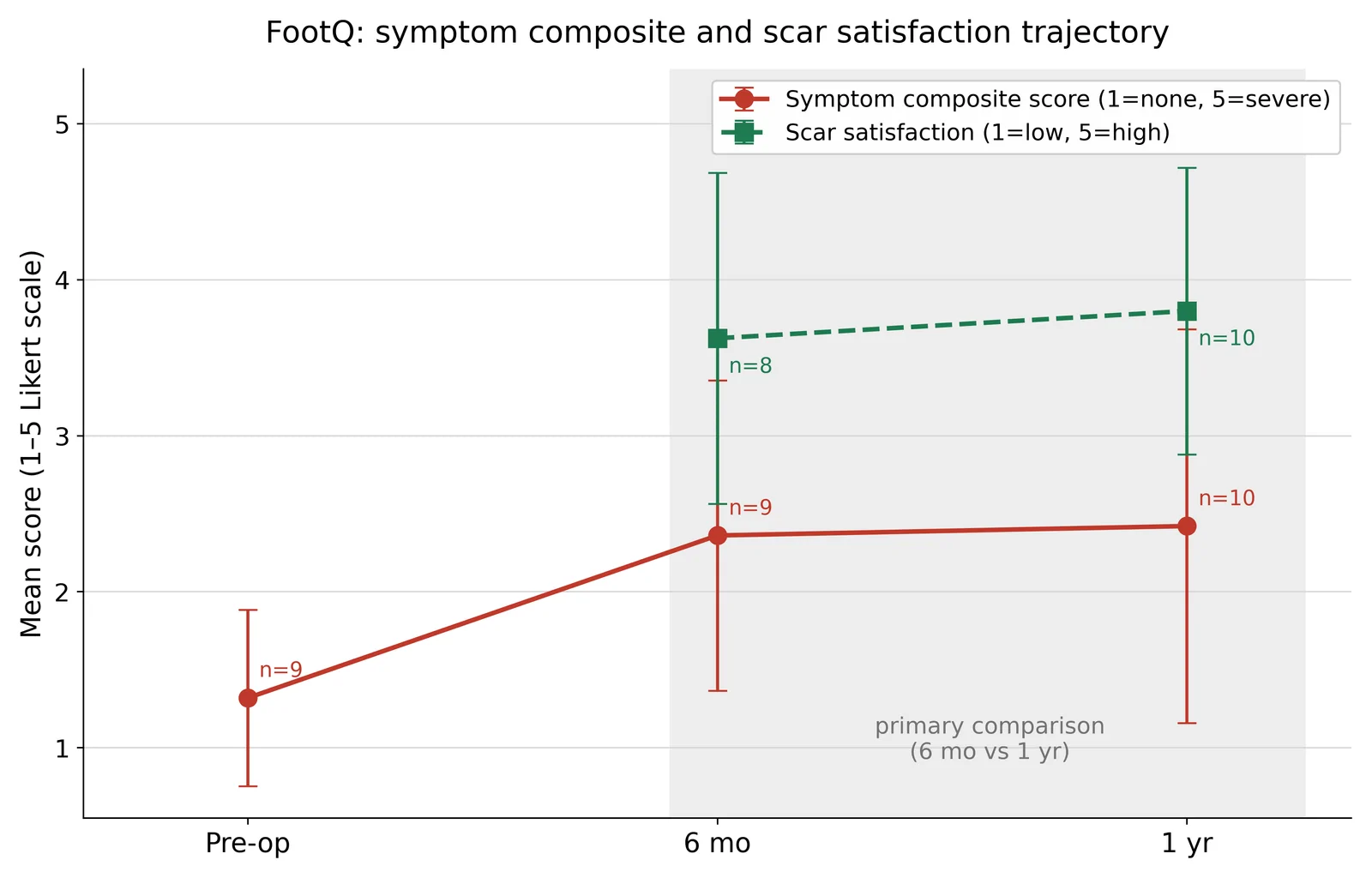
FootQ symptom composite score and scar satisfaction over time. Lines show mean scores at the preoperative, 6-month, and 1-year assessments; error bars are standard deviations. Scar satisfaction is shown only at the two postoperative assessments, because no donor-site scar existed preoperatively and the preoperative response to that item is not interpretable (Section 2.3). Both scales run from 1 to 5—higher symptom scores indicate worse symptoms, and higher satisfaction scores indicate greater satisfaction.

**Table 1:** Patient cohort and donor-site assessment data (n = 10). Altered-sensation (blue) and complete-anesthesia (red) areas were obtained from photographic mapping. In the “Neurotoxic Chemo” column, + indicates that the patient had received neoadjuvant or adjuvant neurotoxic chemotherapy (a taxane-based regimen, with or without platinum) and − indicates that she had not; in the “Side” column, L and R denote a left and a right donor leg, respectively. The donor side was verified against NCS findings where available. Patient 3 had generalized peripheral neuropathy confirmed on NCS; a chemotherapy-induced contribution is possible but unconfirmed, as no preoperative study was available. Measurement uncertainty for the mapped areas is described in Section 2.2.

Pt.	Harvest (cm)	Altered-Sensation (Blue) Area (cm^2^)	Complete-Anesthesia (Red) Area (cm^2^)	Neurotoxic Chemo	Side	NCS SNAP (µV)
1	14	25.2	0	+	L	unrecordable
2	12	19.6	0	+	L	unrecordable
3	26	6.1 (3 zones)	9.6	+	L	unrecordable
4	19	37.2	0	−	L	0.86 (preserved)
5	24	6.4	34.0	−	L	no data
6	24	14.8 (2 zones)	0	−	L	unrecordable
7	23	11.4	18.4	−	L	unrecordable
8	24	32.8 (2 lobes)	0	−	R	2.9 (preserved)
9	24	19.3 (2 zones)	0	+	L	unrecordable
10	24	13.6 (2 zones)	0	+	L	unrecordable

**Table 2:** Descriptive statistics: harvest length and sensory-deficit areas (n = 10).

Variable	Mean	SD	Median	Min	Max	CV (%)
Harvest (cm)	21.4	4.8	24	12	26	22
Altered sensation (blue) (cm^2^)	18.6	10.5	17.1	6.1	37.2	56
Complete anesthesia (red) (cm^2^)	6.2	11.6	0	0	34.0	187
Total (cm^2^)	24.8	9.7	22.4	13.6	40.4	39

**Table 5:** Sural nerve conduction study findings at the final assessment (n = 9; no report was available for Patient 5). All studies were performed 12 months postoperatively except in Patient 8, who was studied at 10 months (Section 2.1).

Pt.	Side	Operated (Donor) Side SNAP	Control Side SNAP	Additional NCS Finding
1	L	unrecordable	1.53 ms/19.4 µV/52.3 m/s	no PNP
2	L	unrecordable	1.69 ms/14.0 µV/50.3 m/s	no PNP
3	L	unrecordable	1.97 ms/6.5 µV/40.6 m/s	generalized sensorimotor axonal-demyelinating PNP (etiology undetermined)
4	L	1.88 ms/0.86 µV/47.9 m/s (preserved, low amplitude)	1.52 ms/20.8 µV/52.6 m/s	—
6	L	unrecordable	1.77 ms/10.1 µV/50.8 m/s	possible L5 radiculopathy (incidental)
7	L	unrecordable	1.76 ms/17.8 µV/51.1 m/s	no PNP
8	R	1.79 ms/2.9 µV/50.3 m/s (preserved, low amplitude)	1.79 ms/20.0 µV/53.1 m/s	no PNP
9	L	unrecordable	1.98 ms/18.5 µV/50.5 m/s	no PNP
10	L	unrecordable	1.74 ms/24.2 µV/51.7 m/s	no PNP

**Table 6:** Semmes–Weinstein monofilament thresholds at the foot and ankle sites over time (mean, SD; range).

Timepoint	n	Foot Site—Mean (SD; Range)	Ankle Site—Mean (SD; Range)
Pre-op	4	1.85 (0.40; 1.65–2.44)	1.65 (0.00; 1.65–1.65)
1 mo	8	2.09 (0.65; 1.65–3.22)	1.94 (0.58; 1.65–3.22)
2 mo	9	2.21 (0.59; 1.65–3.22)	1.97 (0.39; 1.65–2.44)
3 mo	9	2.30 (0.83; 1.65–4.17)	1.97 (0.37; 1.65–2.36)
6 mo	9	2.17 (0.90; 1.65–4.08)	1.98 (0.56; 1.65–3.22)
1 yr	8	1.65 (0.00; 1.65–1.65)	1.65 (0.00; 1.65–1.65)

**Table 7:** Two-point discrimination, operated vs. control leg, by timepoint (4 sites per leg).

Timepoint	Limb	n (Sites)	Normal (%)	Impaired (%)	Anesthesia (%)
Pre-op	Operated	36	56	44	0
Pre-op	Control	36	64	36	0
1 mo	Operated	36	47	50	3
1 mo	Control	36	44	47	8
6 mo	Operated	32	53	44	3
6 mo	Control	32	47	47	6
1 yr	Operated	32	53	47	0
1 yr	Control	32	53	47	0

## Data Availability

The data presented in this study are available on request from the corresponding author owing to privacy and ethical restrictions related to patient confidentiality.

## Supplementary Materials

The following supporting information can be downloaded at https://www.mdpi.com/article/10.3390/medicina62101864/s1: Supplementary Table S1, sensitivity and exploratory analyses of the FootQ symptom composite score; and Supplementary Table S2, FootQ item-level comparison, 6 months vs. 1 year.
