# Supplementary material for: Sural Nerve Graft Donor-Site Morbidity in Nipple–Areola Complex Sensory Reinnervation: A Prospective Cohort Study
Source: Medicina (Kaunas). 2026 Sep 26;62(10):1864. doi: 10.3390/medicina62101864 (PMC13707161; doi:10.3390/medicina62101864)
Supplement: Supplementary file 1 [file medicina-62-01864-s001.zip › medicina-4508960-supplementary.pdf]

Supplementary Materials

Sural Nerve Graft Donor-Site Morbidity in Nipple–Areola Complex Sensory Reinnervation: A Prospective Cohort Study

Jānis Lapiņš, Beatriz Soares Domingues Polita, Linda Kalniņa, Michal Grucki, Dzintars Ozols, Ansis Ģīlis, Arvīds Irmejs, Kristaps Blūms, Gundega Ķauķe, Jānis Gardovskis and Jeļena Maksimenko

Supplementary Table S1. Sensitivity and exploratory analyses of the FootQ symptom composite score (t = paired t-test; W = Wilcoxon signed-rank). Both tests are reported for transparency; as stated in Section 2.6, the non-parametric (Wilcoxon) result is the primary one and the paired t-test is a sensitivity check.

**Comparison**	**n**	**Mean (start → end)**	**p value**	**Effect size**
6 mo vs. 1 yr, composite (all paired)	9	2.36 → 2.57	0.617 (t) / 0.781 (W)	d = 0.17
6 mo vs. 1 yr, composite (excl. one atypical 6-mo response)	8	2.53 → 2.37	0.359 (t) / 0.281 (W)	—
Pre-op vs. 6 mo, composite	8	1.34 → 2.19	0.058 (t) / 0.047 (W)	—
Pre-op vs. 1 yr, composite	9	1.32 → 2.19	0.073 (t) / 0.078 (W)	—
Friedman: pre-op / 6 mo / 1 yr (all)	8	1.34 / 2.19 / 2.32	χ^2 = 7.10, 0.029	—
Friedman: pre-op / 6 mo / 1 yr (excl. atypical response)	7	1.39 / 2.36 / 2.07	χ^2 = 7.69, 0.021	—

Note on the sensitivity rows above: the response excluded in the rows marked “excl. atypical response” / “excl. one atypical 6-mo response” is Patient 4, whose 6-month questionnaire reported a complete absence of donor-site symptoms (composite score 1.00) although she reported marked symptoms at 1 year (composite 4.13) and had the largest mapped deficit area in the cohort (37.2 cm^2). This sensitivity check tests whether that single, internally inconsistent response drives the significance of the overall symptom trajectory. It does not: the Friedman result remains significant whether she is included (χ^2 = 7.10, p = 0.029, n = 8) or excluded (χ^2 = 7.69, p = 0.021, n = 7).

Supplementary Table S2. FootQ item-level comparison, 6 months vs. 1 year. p-values are from the Wilcoxon signed-rank test. Higher scores indicate worse symptoms, except scar satisfaction (*), where higher indicates greater satisfaction.

**Item**	**n**	**Mean 6 mo**	**Mean 1 yr**	**Δ**	**p (Wilcoxon)**
Leg pain	9	2.56	2.89	+0.33	0.750
Numbness	9	2.78	3.00	+0.22	1.000
Numbness-related discomfort	9	2.11	2.22	+0.11	1.000
Mechanical allodynia	9	2.56	2.44	−0.11	1.000

Thermal allodynia	8	2.25	2.75	+0.50	0.750
Hyperesthesia	9	2.44	2.67	+0.22	0.938
Paresthesia	8	2.25	2.50	+0.25	1.000
Stiffness	9	2.11	2.33	+0.22	1.000
Scar satisfaction*	8	3.62	3.88	+0.25	0.500
